# Simultaneous Determination of Escitalopram Impurities including the *R*-enantiomer on a Cellulose tris(3,5-Dimethylphenylcarbamate)-Based Chiral Column in Reversed-Phase Mode

**DOI:** 10.3390/molecules27249022

**Published:** 2022-12-17

**Authors:** Zoltán-István Szabó, Ágnes Bartalis-Fábián, Gergő Tóth

**Affiliations:** 1 Department of Pharmaceutical Industry and Management, George Emil Palade University of Medicine, Pharmacy, Science, and Technology of Targu Mures, Gh. Marinescu 38, 540139 Targu Mures, Romania; 2 Sz-Imfidum Ltd., Lunga nr. 504, 525401 Covasna, Romania; 3 Department of Pharmaceutical Chemistry, Semmelweis University, H-1085 Budapest, Hungary

**Keywords:** citalopram, Lux Cellulose-1, reversed-phase enantioseparation, chemoselectivity, chiral separation

## Abstract

A high-performance liquid chromatographic method was developed for the simultaneous determination of the related substances—three potential synthesis-related chemical impurities and the distomer—of escitalopram. The separation capacity of seven different polysaccharide-type chiral columns, including three amylose-based (Lux Amylose-1, Lux i-Amylose-1, Lux Amylose-2) and four cellulose-based columns (Lux Cellulose-1, Lux Cellulose-2, Lux Cellulose-3, and Lux Cellulose-4) were screened in the polar organic and reversed-phase modes. Lux Cellulose-1, based on cellulose tris(3,5-dimethylphenylcarbamate) as the chiral selector with an acetonitrile-water mixture containing 0.1% diethylamine was identified as the most promising separation system. Using the “one factor at a time” optimization approach, the effect of column temperature, flow rate, and mobile phase constituents on separation performance was evaluated, and the critical resolution values were determined. A U-shaped retention pattern was obtained when plotting the retention factors of the citalopram enantiomers versus the water content of the binary mobile phases on the Lux Cellulose-1 column. A thermodynamic analysis revealed enthalpy-driven enantioseparation in both the polar organic and reversed-phase modes. For further method optimizations, an L9 orthogonal array table was employed. Using the optimized parameters (Lux Cellulose-1 column with 0.1% (*v*/*v*) diethylamine in water/acetonitrile 55/45 (*v*/*v*); 0.8 mL/min flow rate at 25 °C), baseline separations were achieved between all compounds. Our newly developed HPLC method was validated according to the ICH guidelines and its application was tested with a commercially available pharmaceutical formulation. The method proved to be suitable for routine quality control of related substances and the enantiomeric purity of escitalopram.

## 1. Introduction

Escitalopram (*S*-1-[3-(dimethylamino)propyl]f-1-(4-fluorophenyl)-1,3-dihydroisobenzofuran-5-carbonitrile), the *S*-enantiomer of citalopram, is a selective serotonin reuptake inhibitor for the treatment of major depressive disorder or generalized anxiety disorder [1]. Escitalopram presents a greater efficacy and faster onset of action compared to the racemic drug. The lower efficacy of the racemic mixture is due to the inhibition of the antidepressant effect of the *S*-enantiomer by the *R*-enantiomer, possibly via an allosteric interaction with the serotonin transporter [1,2,3]. Determination of the *R*-enantiomer, as a chiral impurity in escitalopram samples, is a regulatory requirement. Moreover, all specified chemical impurities are of interest in the analysis of an active pharmaceutical ingredient. For chiral substances, routinely used in the pharmaceutical industry and in different pharmacopoeial monographs, separate methods are employed for the quantification of achiral (chemical) and enantiomeric impurities. However, methods that facilitate the simultaneous quantification of both chiral and chemical impurities can save valuable time and money. Using a single chiral column in HPLC, or using an appropriate chiral selector in capillary electrophoresis, can unify the analysis of enantiomeric purity and related substances [4,5,6,7,8].

Based on a literature search, several capillary electrophoretic techniques were developed for the enantioselective determination of citalopram, mainly using different cyclodextrins as chiral selectors [9,10,11,12,13,14,15,16]. Sunghtong et al. developed a single capillary electrophoretic method for the determination of citadiol enantiomers and *R*-citalopram in *S*-citalopram samples using a dual cyclodextrin system, containing β-cyclodextrin and sulfated β-cyclodextrin [16]. Citadiol is one of the synthetic intermediaries of (es)citalopram. Despite the many advantages of capillary electrophoresis (greener, cheaper alternative with low analyte and reagent consumption, and fast method development) [17], direct HPLC using chiral stationary phases (CSPs remains the golden standard in this field. This is mainly related to robust instrumentation, generally higher sensitivity, and better scalability [18].

**Figure 1:**
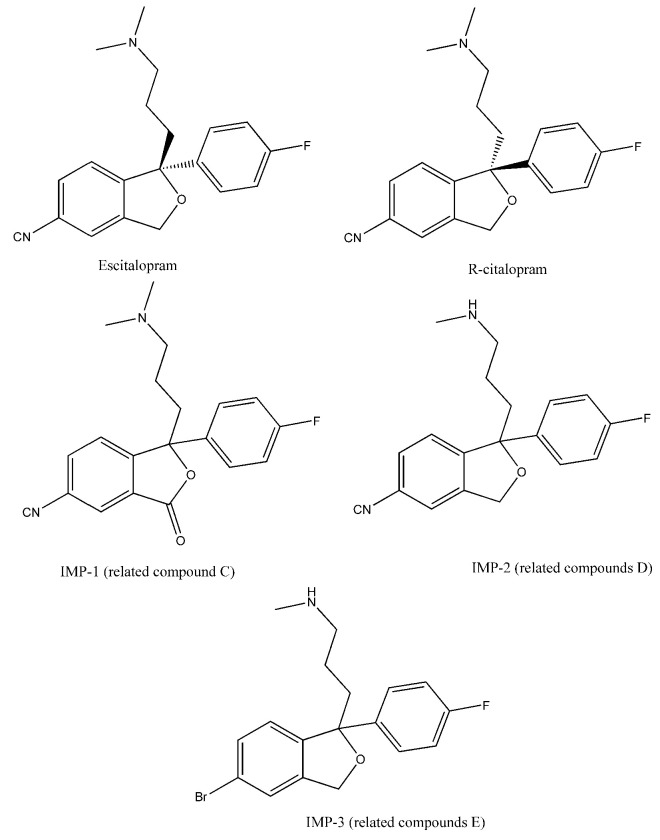
Chemical structure and abbreviations of the compounds used in this study.

Numerous chiral HPLC methods can also be found in the literature, focusing mainly on bioanalytical applications [19,20,21]. Interestingly, only a few publications are focused on pharmaceutical analysis, and only a handful deal with the quantification of the enantiomeric purity of the compound [13,22,23]. Two recent articles determined not only *R*-citalopram, but other in-process impurities such as citadiol or escitalopram *N*-oxide [24,25]. As observed in previous studies, the applied CSPs for the enantioseparation of citalopram show high diversity. Polysaccharide-type stationary phases (Chiralcel OD, Lux Cellulose-2, Kromasil Amycoat) [19,26,27], macrocyclic glycopeptide-type CSPs (Chirobiotic V, V2) [28,29], cyclodextrin-type phases (Cyclobond I 200) [19], and protein-based chiral stationary phases (Chiral AGP) [23] were also used. Many of these earlier methods, especially those employing polysaccharide-type CSPs, were performed using normal-phase chromatography, with toxic eluents, which, if possible, should be avoided in routine quality control. Therefore, an economic yet reliable and fast enantioselective method for the simultaneous analysis of enantiomeric impurity and chemical-related compounds of escitalopram is, urgently needed in pharmaceutical analysis. The aim of the present study was to develop a method for the simultaneous determination of *R*-citalopram, (3*RS*)-6-cyano-3-[3-(dimethylamino)propyl]-3-(4-fluorophenyl)isobenzofuran-1(3H)-one (IMP-1), (1*RS*)-1-(4-fluorophenyl)-1-[3-(methylamino)propyl]-1,3-dihydroisobenzofuran-5-carbonitrile (desmethylcitalopram, IMP-2), and 3-[(1*RS*)-5-bromo-1-4-fluorophenyl)-1,3-dihydroisobenzofuran-1-yl]-*N*,*N*-dimethylpropan-1-amine (IMP-3) (Figure 1), with an acceptance criteria of not more than 0.1% for each impurity. A detailed analysis of chromatographic parameters influencing the separation process, including a thermodynamic analysis of enantioseparation, was also conducted.

## 2. Results and Discussion

### 2.1. Method Development

As a first step in method development, the chiral separation of citalopram enantiomers was attempted, being the most critical part of the given separation problem. Seven different polysaccharide-based CSPs, including amylose-based Lux Amylose-1, Lux i-Amylose-1, and Lux Amylose-2, as well as cellulose-based Lux Cellulose-1, Lux Cellulose-2, Lux Cellulose-3, and Lux Cellulose-4, were tested in the polar organic mode using 0.1% (*v*/*v*) DEA in MeOH, IPA, or ACN as mobile phases, with a 0.6 mL/min flow rate at 25 °C. The aim of these experiments was to select potential chiral selector(s) for further method development. All chromatograms from the scouting phase are depicted in Supplementary Figures S1–S3. Small peak splitting or deformation can be seen in a few cases, which could indicate enantiorecognition. However, enantioseparations with *R*_s_ > 0.5 were observed only on three cellulose-based stationary phases (Table 1).

**Figure 2:**
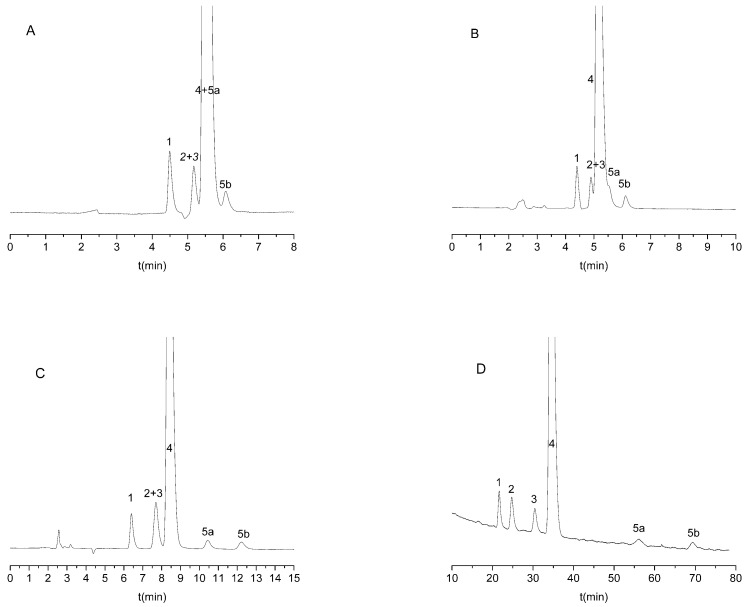
Representative chromatograms on Lux Cellulose-1 column with different acetonitrile water ratio. (**A**) 100% acetonitrile, (**B**) water/acetonitrile 80/20 (*v*/*v*), (**C**) water/acetonitrile 60/40 (*v*/*v*), (**D**) water/acetonitrile 40/60 (*v*/*v*). All mobile phases contain 0.1% diethylamine as basic additive. Other chromatographic parameters: 0.6 mL/min flow rate and 25 °C column temperature. 1: IMP-1; 2: IMP-2; 3: R-citalopram; 4: escitalopram; and 5: IMP-3 (5a is the first eluting enantiomer, while 5b is the second eluting enantiomer of IMP-3).

When using amylose-type CSPs or the methanolic mobile phase, enantiorecognitio was not observed. An appropriate distomer-first elution order was observed only in one case for the Lux Cellulose-1 column with an ACN:DEA 100:0.1 (*v*/*v*) mobile phase;, fortunately, the highest resolution was also observed in this system. This separation system was selected for further method development, even though some of the impurities were found to elute with citalopram enantiomers (Figure 2A). To increase retention of the analytes, the addition of water to the mobile phase was attempted. Increasing the water content of the mobile phase improved both the enantio- and chemoselectivity of the method (Figure 2A–D). The addition of more than 50% water to ACN resulted in the baseline separation of all compounds (Figure 2D).

All three chemical impurities were available as racemates; however, we did not aim for the chiral separation of these enantiomers. We aimed instead to achieve the enantioseparation of citalopram and the separation of the chemical impurities in one single run. As can be observed in the case of IMP-3, the individual enantiomers were also separated, and they did not interfere with the determination of the analytes.

**Figure 3:**
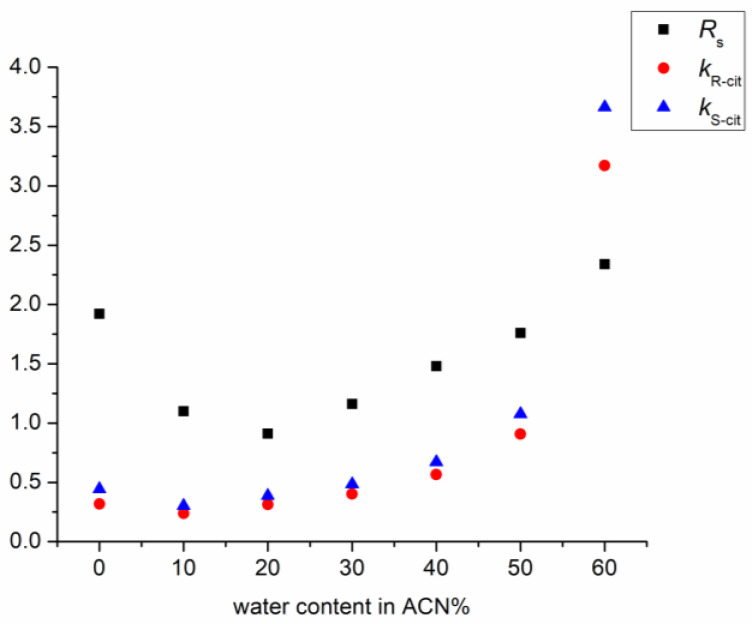
Plots of the retention and resolution factors as a function of the water content in acetonitrile on Lux Cellulose-1 column. (Chromatographic conditions: mobile phase 0.1% DEA in the indicated eluent composition, flow rate: 0.6 mL/min; column temperature: 25 °C).

Figure 3 shows the effect of the water content in the mobile phase on the retention and enantioresolution of citalopram enantiomers using Lux Cellulose-1 CSP. A U-shape retention and resolution profile was observed, which is typical for mixed-mode columns. In the first section of the retention curve, until 20% of water content in the mobile phase was reached, a HILIC-like behavior was observed; an increase in the water content of the mobile phase led to decreased retention. The transition from HILIC to the reversed-phase mode was observed at a water content of 20%. When using more than 20% water in the mobile phase, both the retention factor and resolution began to increase. Similar results were also described by other research groups using polysaccharide-based CSPs [30,31].

To find the optimal parameter ranges, first, a “one factor at a time” screening approach was applied, tracking the obtained critical resolution values, within the following chromatographic parameter ranges: column temperature of 10–40 °C, flow rate between 0.5 and 1 mL/min, and DEA content between 0% and 0.15%. Adding DEA as a basic additive to the mobile phase was necessary for enantioseparation; however, a concentration of higher than 0.1% did not have a significant influence on separation performance.

The method was further fine-tuned using an L9 orthogonal array table. The chromatographic parameter ranges were restricted based on the previous screening experiments (Table 2). Both critical resolutions, *R*_s2_ (resolution between IMP-2 and *R*-citalopram) and *R*_s3_ (resolution between *R*-citalopram and *S*-citalopram), were selected as response values. The chart of the L9 orthogonal array table and the critical resolution values obtained at each experimental run are presented in Table 2.

In order to analyze the impact of a particular factor on the enantioseparation, a range analyses were applied. The average *R*_s_ values were calculated for each of the three levels of a factor (K1–K3), i.e., for *R_s2_*, the K1 value of 1.33 was the average *R_s2_* value obtained at 15 °C (experiments 1–3) (Table 2) The range values (R) are the differences between the maximal and minimal K values, thus providing information about the impact of each factor on *R*_s2_ and *R_s_*_3_; the higher the range value, the greater the impact. As can be observed, the most important factor to consider is the water content of the mobile phase, as it presents the highest range value. The other two parameters have a smaller effect on the critical resolution values. Based on the results, a higher water content resulted in higher resolution values; however, it was also accompanied by a longer analysis time. Using 60 % water in the mobile phase, an analysis time of less than 30 min was not possible; therefore, a mobile phase consisting of water/acetonitrile 55/45 (*v*/*v*) with 0.1% DEA was selected for further studies. 

**Figure 4:**
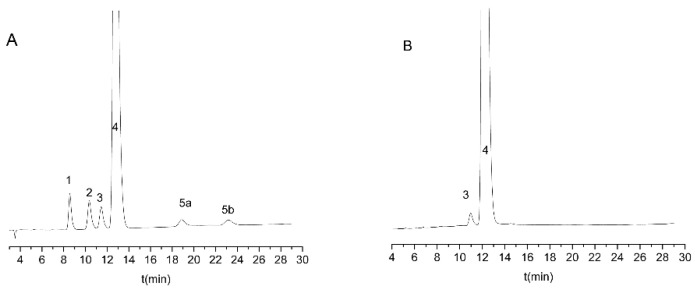
Representative chromatograms obtained during method optimization and application. (**A**) Solution of escitalopram sample spiked with 0.1% impurities. (**B**) Solution of escitalopram 10 mg tablet. Experimental conditions: Lux Cellulose-1 column with 0.1% (*v*/*v*) diethylamine in water/acetonitrile 55/45 (*v*/*v*), 0.8 mL/min flow rate at 25 °C.

Regarding temperature, 25 °C was the best value for both investigated resolution values, as it offered the highest critical resolutions in the shortest analysis time. By analyzing the effect of the flow rate, it can be observed that a lower flow rate resulted in a higher resolution in the case of *R_s_*_2,_; however, a higher flow rate was accompanied by a higher resolution value for *Rs*_3_. Considering the combined effect on both critical resolution values, a 0.8 mL/min flow rate was selected as the optimal rate. Based on these values, the Lux Cellulose-1 column at 25 °C, with a mobile phase consisting of 0.1% (*v*/*v*) diethylamine in water/acetonitrile 55/45 (*v*/*v*), and delivered with a flow rate of 0.8 mL/min, was selected as the final method. Under these circumstances, all analytes were baseline separated within 30 min; a representative chromatogram is depicted in Figure 4A.

### 2.2. Method Validation and Application

Validation of the optimized method was performed according to the International Council for Harmonization guidelines (ICH) Q2 (R1) for all related substances and for *R*-citalopram as a chiral impurity with respect to sensitivity, linearity, accuracy, and precision. The limit of detection (LOD) and the limit of quantification (LOQ) were calculated based on signal-to-noise ratios of 3:1 and 10:1 for the LOD and LOQ, respectively. The validation data are summarized in Table 3. The linearity of the method was evaluated at eight concentration levels for all impurities and calibration plots are provided with peak areas plotted against corresponding concentrations (expressed in μg/mL). The correlation coefficients were determined by performing a linear least squares regression analysis, and it was higher than 0.9987 in all cases. Moreover, for all impurities, it was observed that 95% confidence intervals of the y-intercepts included zero and random distributions of the residuals. The accuracy and precision were analyzed by performing intra- (repeatability) and inter-day evaluations (two consecutive days) on three concentration levels for all impurities (low, medium, high) covering the linearity range, with each solution being injected five times. For all impurities, the accuracy (expressed in average recovery%) ranged from 98.32% to 101.59%, with less than 1% standard deviation. Intraday precision (expressed as RSD%) was in the range of 0.09–1.11%, while RSD for intermediate precision was below 1.35%. Based on the results obtained during the validation, the method proved to be sensitive, linear, accurate, and precise for the determination of the selected impurities in the escitalopram.

The optimized and validated method was applied for the analysis of real samples by means of film-coated tablets with a nominal content of 10 mg escitalopram in the form of escitalopram oxalate. Representative chromatograms are shown in Figure 4B. From the impurities only, *R*-citalopram can be identified in the sample, and the quantity of other impurities is under 0.05%. The content of *R*-citalopram was 0.71 ± 0.01%, which meets the requirements of the limit stated both in the United States Pharmacopoeia (not higher than 3%), and in the European Pharmacopoeia (not higher than 2%).

### 2.3. Thermodynamic Study

Thermodynamic study is a useful and widely applied method for the investigation of chiral recognition mechanisms [32,33,34]. Chromatographic runs performed at different temperatures provided an opportunity to compare the thermodynamic parameters in the reversed phase and polar organic mode for the enantioseparation of citalopram. To reveal the effect of temperature on retention and selectivity, the classical Van’t Hoff analysis was applied. Although this approach is often used due to its simplicity, it does not distinguish between enantioselective and non-enantioselective interactions; thus, the thermodynamic values obtained herein are apparent [35].

The differences in the change of standard enthalpy Δ*(*ΔH°) and standard entropy Δ*(*Δ*S°)* for the enantiomeric pair, in the reversed-phased and polar organic mode on the Lux Cellulose-1 column was calculated by plotting ln*α* vs. 1/*T*, based on the following equation:

(1) $$\ln\alpha =-\frac{\Delta \Delta H^{{}^{\circ}}}{RT}+\frac{\Delta \Delta S^{{}^{\circ}}}{R}$$

where *R* stands for universal gas constant, *T* is the temperature, expressed in Kelvin and α is the selectivity factor.

The iso-enantioselective temperatures (*T_iso_*) were also calculated as follows:

*T_iso_* = Δ(ΔH°)/Δ(ΔS°) (2)

*T_iso_* represents the compensation between enthalpy and entropy, and the two enantiomers coelute and no separation is achieved. For temperatures of above *T_iso_*, the separation is entropy-controlled, whereas below *T_iso_* it is enthalpy-controlled. When changing the temperature from entropy-controlled to enthalpy-controlled, enantiomer order reversal is expected. *Q* values were used to visualize the relative contribution of enthalpy and entropy terms to the free energy of adsorption, as follows:

*Q* = Δ*(*ΔH°)/TxΔ(Δ*S°*)_298K_ (3)

Thermodynamic data are summarized in Table 4.

As can be observed, retention factors and selectivity decreased with increasing temperature and positive *Q* values were obtained in both cases, which means that enantioseparation was mainly driven by enthalpic contributions. It can also be seen that thermodynamic parameters such as Δ(ΔH°), Δ(Δ*S°*), *Tx*Δ(Δ*S°*), and Δ(Δ*G°*) were lower in the polar organic mode than in the reversed phase mode in our case. Moreover, the *T_iso_* value was relatively low in both modes; however, a lower value was observed for the polar organic mode.

## 3. Materials and Methods

### 3.1. Materials

Escitalopram oxalate and racemic citalopram HBr were ordered from Sigma-Aldrich Hungary (Budapest, Hungary). Chemical impurities such as (3*RS*)-6-cyano-3-[3-(dimethylamino)propyl]-3-(4-fluorophenyl)isobenzofuran-1(3*H*)-one, (1*RS*)-1-(4-Fluorophenyl)-1-[3-(methylamino)propyl]-1,3-dihydroisobenzofuran-5-carbonitrile hydrochloride (Desmethylcitalopram Hydrochloride), and 3-[(1*RS*)-5-Chloro-1-(4-fluorophenyl)-1,3-dihydroisobenzofuran-1-yl]-N,N-dimethylpropan-1-amine oxalate were obtained from LGC GmbH (Luckenwalde, Germany). HPLC-grade MeOH, ACN, IPA, and diethylamine were purchased from Merck (Darmstadt, Germany). The deionized water was prepared with a Milli-Q Direct 8 Millipore system. Lux Cellulose-1 (150 × 4.6 mm; particle size: 5 µm) [based on cellulose tris(3,5-dimethylphenylcarbamate)], Lux Cellulose-2 (150 × 4.6 mm; particle size: 5 µm) [based on cellulose tris(3-chloro-4-methylphenylcarbamate)], Lux Cellulose-3 (150 × 4.6 mm; particle size: 5 µm) [based on cellulose tris(4-methylbenzoate)], Lux Cellulose-4 (150 × 4.6 mm; particle size: 5 µm) [based on cellulose tris(4-chloro-3-methylphenylcarbamate)], Lux Amylose-1 (150 × 4.6 mm; particle size: 5 µm) [based on amylose tris(3,5-dimethylphenylcarbamate)], Lux i-Amylose-1 (150 × 4.6 mm; particle size: 5 µm) [based on amylose tris(3,5-dimethylphenylcarbamate)], and Lux Amylose-2 (Am2) (150 × 4.6 mm; particle size: 5 µm) [based on amylose tris(5-chloro-2-methylphenylcarbamate) were all obtained from Phenomenex (Torrance, CA, USA). Escigen 10 mg film-coated tablets were purchased from a local pharmacy in Budapest, Hungary.

### 3.2. LC-UV Analysis

Chromatographic experiments were performed with a Jasco HPLC system with of a PU-2089 plus quaternary pump, AS-4050 autosampler, MD-2010 diode array detector, and a Jetstream 2 Plus thermostat. JASCO ChromNAV software was used for instrument control and data analysis. Unless stated otherwise, separations were performed at 25 °C using a 0.6 mL/min flow rate. UV detection was performed at 230 nm. In the screening phase, alcohols—MeOH and IPA—and ACN were used, and the sample contained only enantio-enriched citalopram.

The developed method was validated for the simultaneous analysis of related substances and the enantiomeric purity of escitalopram. MeOH was used as the solvent for the preparation of stock solutions throughout the study, and it was further diluted with the appropriate mobile phases when necessary. The final test solution of escitalopram used for simultaneous achiral and enantiomeric purity testing was about 4000 µg mL^−1^. All impurity level percentages were reported at this concentration. An injection volume of 3 μL was used.

For preparation of sample solutions, twenty film-coated tablets were weighted, then ground and mixed in a mortar. In a 10 mL volumetric flask, MeOH was added to an accurately weighted portion of the tablet powder, corresponding to about 40 mg escitalopram. Subsequently, the suspension was sonicated for 30 min and centrifuged for 5 min, applying 4000 rpm (Sartorius 2–16 P benchtop centrifuge, Goettingen, Germany). The clear supernatant was filtered through a 0.22 μm pore size PVDF syringe filters (FilterBio membrane Co., LTD, Nantong, China).

## 4. Conclusions

A single-run chemo- and enantioselective method was developed for the determination of related substances of escitalopram, including its enantiomeric pair on a polysaccharide column. The initial screening phase was based on the determination of the chiral discrimination capabilities of polysaccharide-type chiral stationary phases in the polar organic and reverse-phase modes, as the most critical part of the method. Lux Cellulose-1, based on cellulose tris(3,5-dimethylphenylcarbamate), was identified as the most promising column, using a mobile phase consisting of water-acetonitrile mixtures containing 0.1% diethylamine as a basic modifier. Upon tracking the retention times of the enantiomers as a function of the water content of the mobile phase, U-shaped retention curves were obtained, revealing a gradual transition from HILIC-like to typical reverse-phase behavior. The thermodynamic characterization, using the classical Van’t Hoff analysis, revealed an enthalpy-driven separation. The chemo- and enantioselectivity of the method was further fine-tuned using an L9 orthogonal array table and provided a baseline separation of all analytes under optimized conditions (Lux Cellulose-1 column with a mobile phase consisting of water/acetonitrile 55/45 (*v*/*v*), containing 0.1% (*v*/*v*) diethylamine). The method was subsequently validated according to the ICH guidelines and applied on real, commercial samples containing escitalopram. The single-run, chemo-, and enantioselective method could offer a valuable cost and time-saving alternative to the presently often-applied approach using a chiral- and a separate achiral chromatographic system. 

## Figures and Tables

**Table 1:** Chromatographic data obtained during the preliminary study related to retention factor of the second-eluting enantiomer (*k*_2_), resolution (*R*_s_), and elution order for the chromatographic systems where enantiorecognition was observed.

Column Type	Mobile Phase	*k*_2_	*R*_s_	Elution Order
Lux Cellulose-3	IPA:DEA 100:0.1 (*v*/*v*)	0.58	0.6	*S < R*
Lux Cellulose-2	ACN:DEA 100:0.1 (*v*/*v*)	0.72	1.0	*S < R*
Lux Cellulose-1	ACN:DEA 100:0.1 (*v*/*v*)	0.94	1.2	*R < S*

**Table 2:** L9 orthogonal array table used for method optimization, with the obtained critical resolution values.

Experimental No.	Temperature (°C)	Flow (mL/min)	Water Content in ACN (%)	*R*_s2_	*R*_s3_
1	15	0.7	50	0.72	0.98
2	15	0.8	55	1.25	1.33
3	15	0.9	60	2.02	1.69
4	25	0.7	55	1.66	1.23
5	25	0.8	60	1.88	1.75
6	25	0.9	50	0.78	1.05
7	35	0.7	60	1.89	1.67
8	35	0.8	50	0.85	0.91
9	35	0.9	55	1.28	1.29
Results for *R*_s2_					
K1	1.33	1.42	0.78		
K2	1.44	1.33	1.40		
K3	1.34	1.36	1.93		
R	0.10	0.09	1.15		
Results for *R_s_*_3_					
K1	1.34	1.30	0.98		
K2	1.34	1.33	1.28		
K3	1.29	1.34	1.70		
R	0.05	0.04	0.72		

**Table 3:** Summary of data obtained during method validation for the simultaneous determination of related substances and enantiomeric purity.

Parameter	Level	IMP-1	IMP-2	*R*-cit	IMP-3a	IMP-3b
Range (μg/mL)		2–40	2–40	2–40	4–40	4–40
Range (%)		0.05–1	0.05–1	0.05–2	0.1–1	0.1–1
r^2^		0.9990	0.9989	0.9995	0.9987	0.9985
LOD (μg/mL)		0.51	0.60	0.60	1.20	1.20
LOQ (μg/mL)		1.7	2.0	2.0	4.0	4.0
Accuracy	I. (4 μg/mL)	100.42	100.25	99.12	98.32	98.36
	II. (16 μg/mL)	98.99	99.61	100.49	100.45	101.0
	III. (32 μg/mL)	99.58	101.59	99.58	101.12	99.45
Intraday precision	I. (4 μg/mL)	0.61%	0.55%	0.75%	1.11%	0.89%
	II. (16 μg/mL)	0.65%	0.09%	0.43%	0.42%	0.75%
	III. (32 μg/mL)	0.54%	0.13%	0.80%	0.51%	0.33%
Intermediate precision	I. (4 μg/mL)	0.27%	0.74%	0.30%	1.01%	1.35%
	II. (16 μg/mL)	0.08%	0.12%	0.45%	0.42%	0.35%
	III. (32 μg/mL)	0.05%	0.33%	0.22%	0.23%	0.32%

**Table 4:** Thermodynamic parameters, Δ(ΔH°), Δ(Δ*S°*), *Tx*Δ(Δ*S°*), Δ(Δ*G°*), Van’t Hoff equation, correlation coefficients and *Q* values.

System/Mode	Van’t Hoff Equations	r^2^	−Δ(ΔH°) (kJ mol^−1^)	−Δ(Δ*S°*) (J mol^−1^K^−1^)	−*Tx*Δ(Δ*S°*)_298_*_K_* (kJ mol^−1^)	−Δ(Δ*G°*)_298_*_K_* (kJ mol^−1^)	*T*_(_*_iso_*_)_ (°C)	*Q*
Reversed phase *	Lnα = 148.66x − 0.3642	0.9988	1.2	3.0	0.9	0.3	135	1.4
Polar organic mode **	Lnα = 586.8x − 1.707	0.9989	4.9	14.2	4.2	0.6	70.8	1.2

* Lux Cellulose-1 column with 0.1% (*v*/*v*) diethylamine in water/acetonitrile 55/45 (*v*/*v*), 0.8 mL/min flow rate. ** Lux Cellulose-1 column with 0.1% (*v*/*v*) diethylamine in 100% acetonitrile, 0.8 mL/min flow rate.

## Data Availability

Not applicable.

## Supplementary Materials

The following supporting information can be downloaded at: https://www.mdpi.com/article/10.3390/molecules27249022/s1, Figure S1: Chromatograms from the scouting phase using 0.1% (*v*/*v*) DEA in MeOH as mobile phase with 0.6 mL min^−1^ flow rate at 25 °C. A: Lux Amylose-1, B: Lux Amylose-2, C: Lux i-amylose-1, D: Lux Cellulose-1, E: Lux Cellulose-2, F: Lux Cellulose-3, G: Lux Cellulose-4; Figure S2: Chromatograms from the scouting phase using 0.1% (*v*/*v*) DEA in 2-propanol as mobile phase with 0.6 mL min^−1^ flow rate at 25 °C. A: Lux Amylose-1, B: Lux Amylose-2, C: Lux i-amylose-1, D: Lux Cellulose-1, E: Lux Cellulose-2, F: Lux Cellulose-3, G: Lux Cellulose-4; Figure S3: Chromatograms from the scouting phase using 0.1% (*v*/*v*) DEA in acetonitrile as mobile phase with 0.6 mL min^−1^ flow rate at 25 °C. A: Lux Amylose-1, B: Lux Amylose-2, C: Lux i-amylose-1, D: Lux Cellulose-1, E: Lux Cellulose-2, F: Lux Cellulose-3, G: Lux Cellulose-4.
