# Supplementary material for: Simultaneous Determination of Escitalopram Impurities including the R-enantiomer on a Cellulose tris(3,5-Dimethylphenylcarbamate)-Based Chiral Column in Reversed-Phase Mode
Source: Molecules. 2022 Dec 17;27(24):9022. doi: 10.3390/molecules27249022 (PMC9783879; doi:10.3390/molecules27249022)
Supplement: Supplementary file 1 [file molecules-27-09022-s001.zip › molecules-2090777-supplementary.pdf]

SUPPLEMENTARY INFORMATION

Simultaneous determination of escitalopram impurities including the *R*-enantiomer on a cellulose tris(3,5-dimethylphenylcarbamate)-based chiral column in reversed-phase mode

Zoltán-István Szabó[1,2*], Ágnes Bartalis-Fábián[1], Gergő Tóth[3]

[1] Faculty of Pharmacy, George Emil Palade University of Medicine, Pharmacy, Science, and Technology of Targu Mures, Gh. Marinescu 38, Targu Mures, Romania

[2] Sz-imfidum Ltd., Lunga, Romania

[3] Department of Pharmaceutical Chemistry, Semmelweis University, Budapest H-1085, Hungary

***Corresponding author**:

Zoltán-István Szabó, PhD

Tel: +40744231522

Email address: zoltan.szabo@umfst.ro

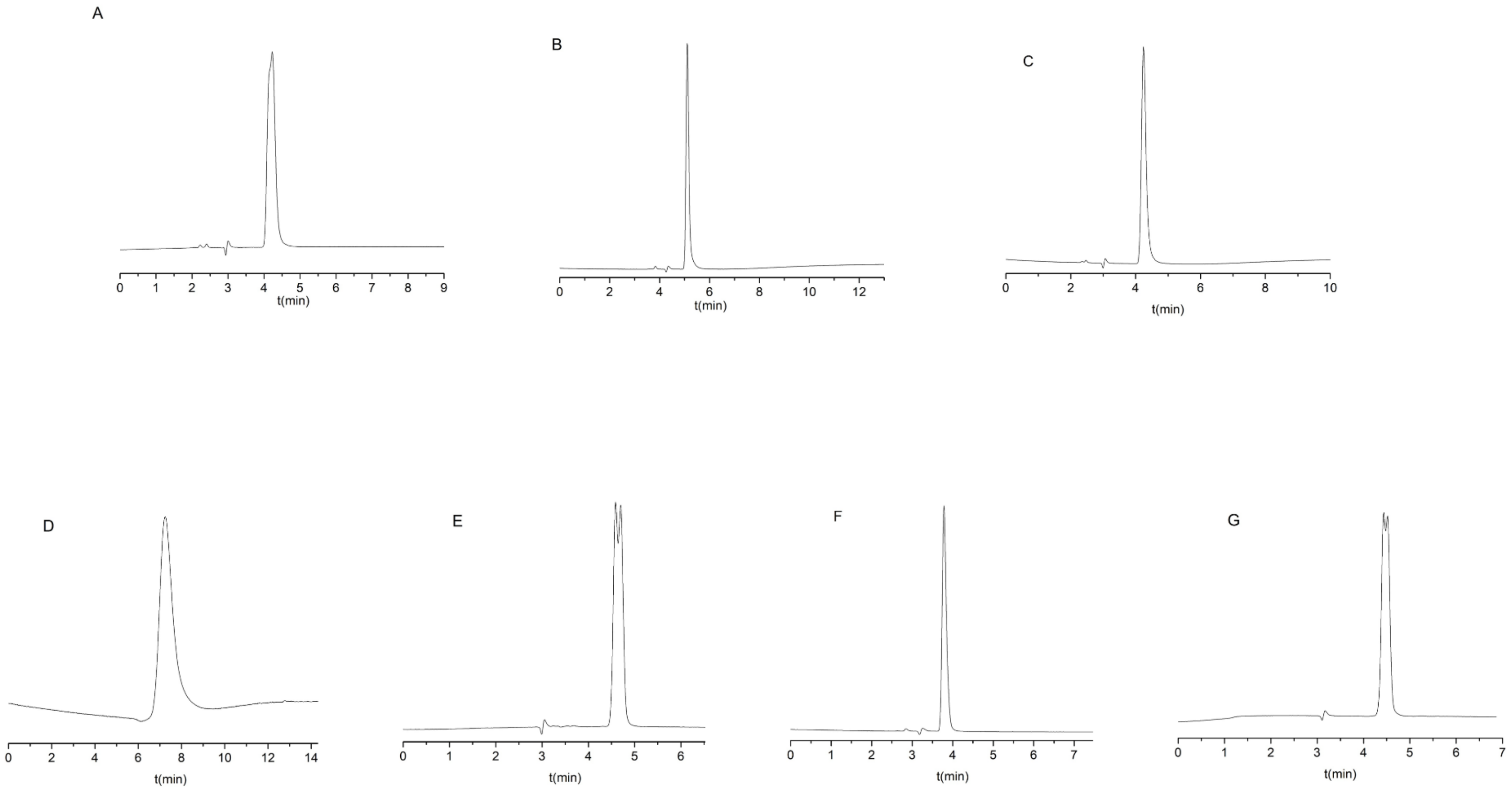

Figure S1 Chromatograms from the scouting phase using 0.1% (v/v) DEA in MeOH as mobile phase with 0.6 mL min^{-1} flow rate at 25 °C. A: Lux Amylose-1, B: Lux Amylose-2, C: Lux i-amylose-1, D: Lux Cellulose-1, E: Lux Cellulose-2, F: Lux Cellulose-3, G: Lux Cellulose-4

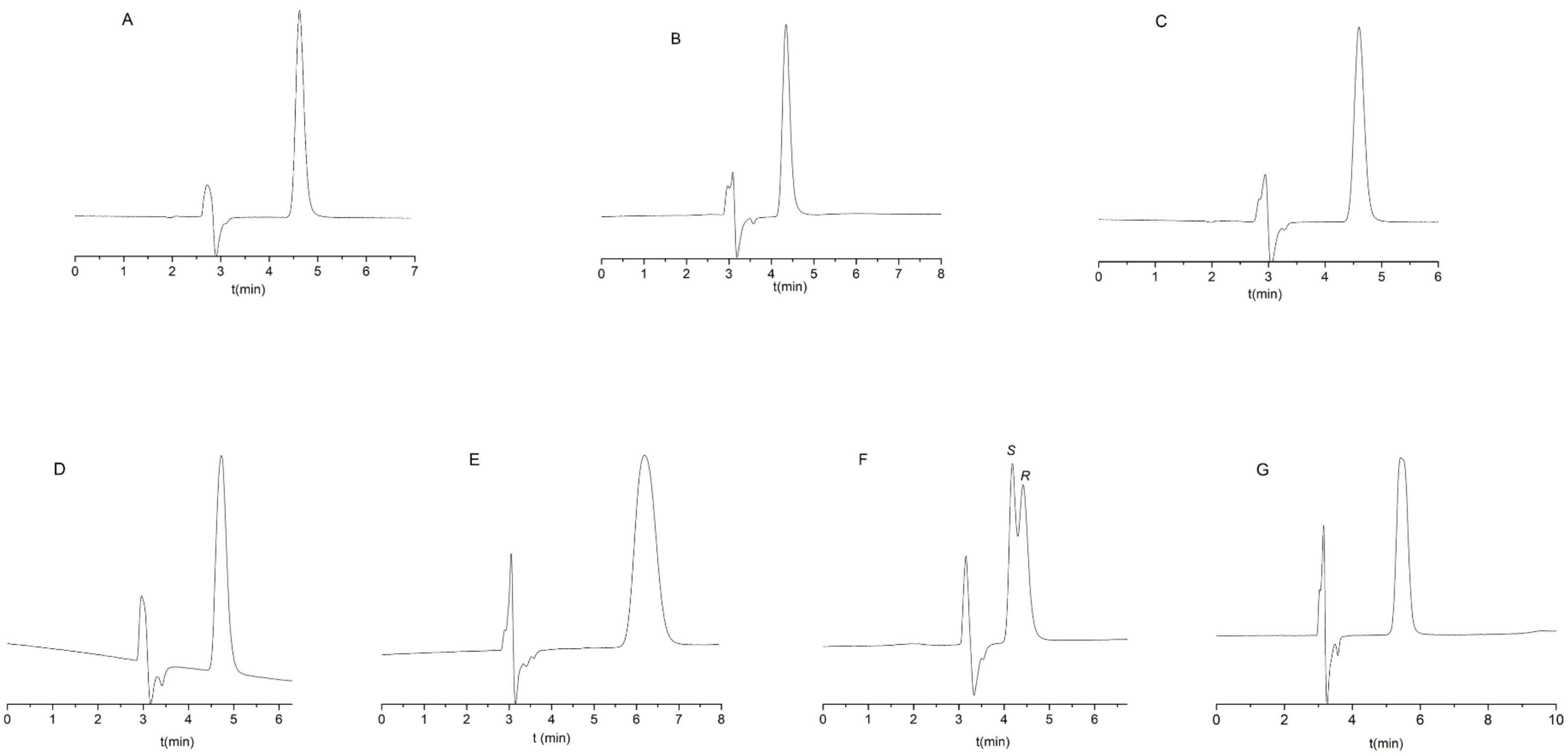

Figure S2 Chromatograms from the scouting phase using 0.1% (v/v) DEA in 2-propanol as mobile phase with 0.6 mL min^{-1} flow rate at 25 °C. A: Lux Amylose-1, B: Lux Amylose-2, C: Lux i-amylose-1, D: Lux Cellulose-1, E: Lux Cellulose-2, F: Lux Cellulose-3, G: Lux Cellulose-4

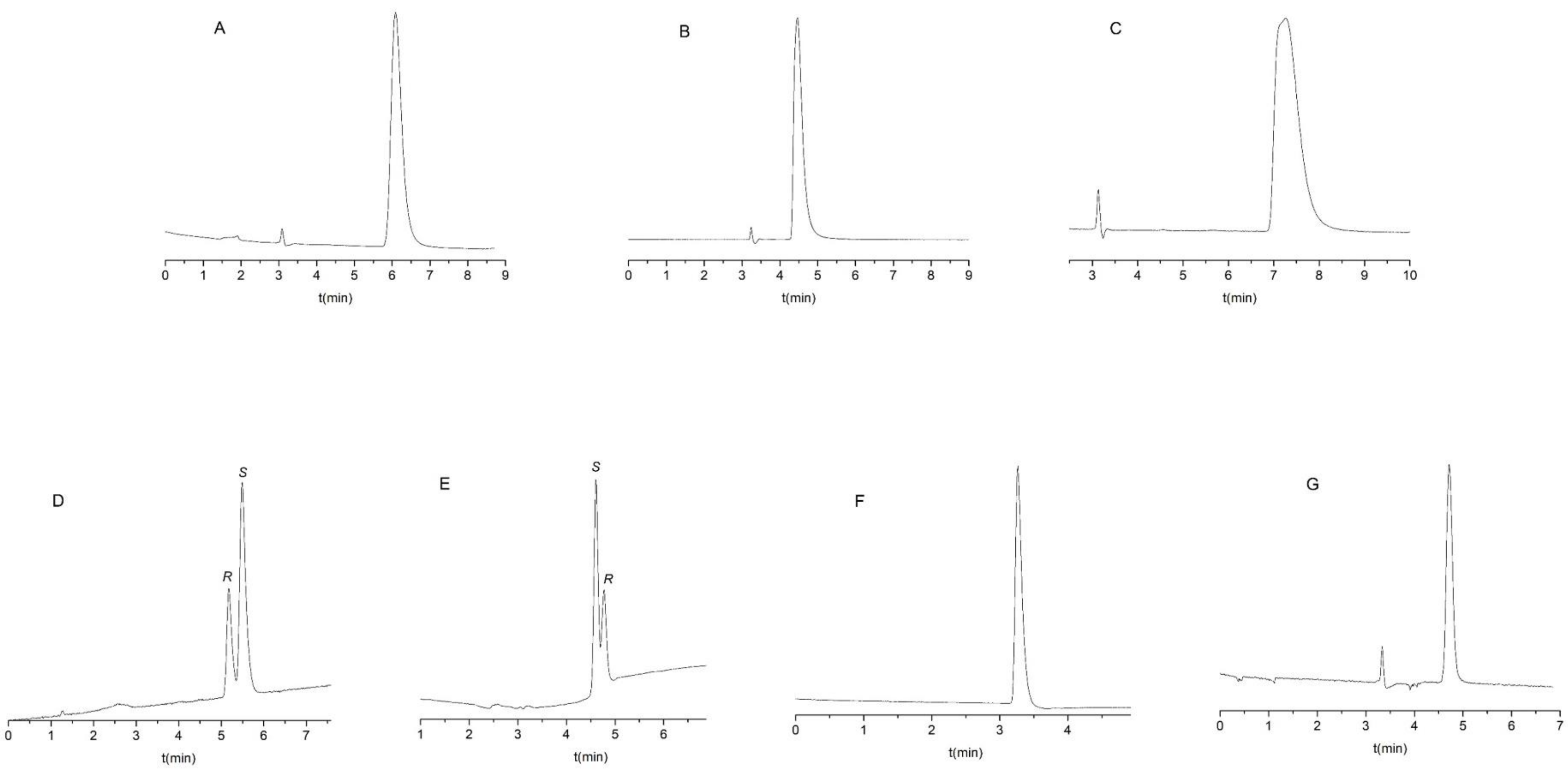

Figure S3 Chromatograms from the scouting phase using 0.1% (v/v) DEA in acetonitrile as mobile phase with 0.6 mL min^{-1} flow rate at 25 °C. A: Lux Amylose-1, B: Lux Amylose-2, C: Lux i-amylose-1, D: Lux Cellulose-1, E: Lux Cellulose-2, F: Lux Cellulose-3, G: Lux Cellulose-4
